# Modulation of MicroRNAs as a Potential Molecular Mechanism Involved in the Beneficial Actions of Physical Exercise in Alzheimer Disease

**DOI:** 10.3390/ijms21144977

**Published:** 2020-07-14

**Authors:** Alex Cleber Improta-Caria, Carolina Kymie Vasques Nonaka, Bruno Raphael Ribeiro Cavalcante, Ricardo Augusto Leoni De Sousa, Roque Aras Júnior, Bruno Solano de Freitas Souza

**Affiliations:** 1 Post-Graduate Program in Medicine and Health, Faculty of Medicine, Federal University of Bahia, Bahia 40110-909, Brazil; aleximprotacaria@gmail.com (A.C.I.-C.); roque.aras@uol.com.br (R.A.J.); 2 University Hospital Professor Edgard Santos, Bahia 40110-909, Brazil; 3 Center for Biotechnology and Cell Therapy, São Rafael Hospital, Bahia 40110-909, Brazil; carolina.nonaka@hsr.com.br (C.K.V.N.); bruno.cavalcante@hsr.com.br (B.R.R.C.); 4 D’Or Institute for Research and Education (IDOR), Rio de Janeiro 20000-000, Brazil; 5 Gonçalo Moniz Institute, Oswaldo Cruz Foundation (FIOCRUZ), Bahia 40110-909, Brazil; 6 Physiological Science Multicentric Program, Federal University of Valleys´ Jequitinhonha and Mucuri, Minas Gerais 30000-000, Brazil; ricardoaugustoleonidesousa@gmail.com

**Keywords:** Alzheimer disease, microRNAs, physical exercise

## Abstract

Alzheimer disease (AD) is one of the most common neurodegenerative diseases, affecting middle-aged and elderly individuals worldwide. AD pathophysiology involves the accumulation of beta-amyloid plaques and neurofibrillary tangles in the brain, along with chronic neuroinflammation and neurodegeneration. Physical exercise (PE) is a beneficial non-pharmacological strategy and has been described as an ally to combat cognitive decline in individuals with AD. However, the molecular mechanisms that govern the beneficial adaptations induced by PE in AD are not fully elucidated. MicroRNAs are small non-coding RNAs involved in the post-transcriptional regulation of gene expression, inhibiting or degrading their target mRNAs. MicroRNAs are involved in physiological processes that govern normal brain function and deregulated microRNA profiles are associated with the development and progression of AD. It is also known that PE changes microRNA expression profile in the circulation and in target tissues and organs. Thus, this review aimed to identify the role of deregulated microRNAs in the pathophysiology of AD and explore the possible role of the modulation of microRNAs as a molecular mechanism involved in the beneficial actions of PE in AD.

## 1. Introduction

Alzheimer disease (AD) is the leading cause of dementia, being defined as a progressive neurodegenerative disease with neuropathological hallmarks: Neurofibrillary tangles and beta-amyloid plaques [1]. Neurofibrillary tangles are formed by hyperphosphorylated Tau protein, which loses its physiological role in promoting microtubule stability in neurons [2,3]. Plaques are aggregates of beta amyloid (Aβ) oligomers, which are highly toxic to the central nervous system (CNS) and promote neuroinflammation [4,5]. Considering that AD’s prevalence is expected to grow, it is crucial to increase pathophysiological knowledge, to identify novel biomarkers for early diagnosis, and to develop direct therapeutic interventions to slow disease progression.

Physical exercise (PE) has been reported to reduce memory loss [6], and sedentarism has been recognized as a modifiable risk factor for the development of AD [7]. The results of a recent meta-analysis indicate that different PE programs have a beneficial impact on patients with dementia, leading to significant improvements in cognitive function, memory, and ability to perform daily activities [8]. The mechanisms involved in the benefits promoted by PE in AD remain not fully understood.

It is known that PE has antioxidant [9] and anti-inflammatory actions that contribute to neuroprotection and cognitive improvements seen in animal models and AD patients [10]. In AD mouse models, PE modulated neuroinflammation, improved memory [11,12,13], reduced neuronal death, and increased the expression of antioxidant enzymes [14]. Moreover, PE has also been reported to attenuate the accumulation of Aβ plaques in the brain, increase hippocampal volume, and improve behavior in a Tg2576 AD mouse model [15].

PE modulates the profile of microRNA expression detected in body fluids, tissues, and organs. The hypothesis that modulation of microRNA expression by PE is mechanistically linked to the improvements reported in cognitive function in the context of AD, however, has not been sufficiently explored to date [16,17,18]. MicroRNAs (miRNAs) are small, single-stranded non-coding RNAs that play key roles in the post-transcriptional regulation of gene expression [19]. MiRNAs bind to the 3′-untranslated region (3′UTR) of target mRNAs [20], leading to degradation, deadenylation, or inhibition of translation [21].

A single miRNA can regulate different targets, and it is estimated that miRNAs regulate approximately 60% of all human protein coding genes by post-transcriptional mechanisms [22]. Therefore, miRNAs are involved in several biological processes, including apoptosis [23], cell metabolism, homeostasis [24], differentiation [25], cell cycle [26], growth [27], cell survival [28], cell proliferation, and migration [29]. In addition, the involvement of miRNAs has also been demonstrated in pathological processes of different diseases, such as heart failure [30], arterial hypertension [31], type 2 diabetes mellitus, obesity [32], Chagas disease cardiomyopathy [33], and AD [34].

In this review, we aimed to evaluate the evidence supporting the role of deregulated miRNA profiles in the pathogenesis of AD and the potential beneficial actions of PE-induced miRNAs in physiological brain functions. Overlaps in the miRNAs reported to be deregulated in AD and induced by PE were also evaluated and discussed.

## 2. Biogenesis of MiRNAs

MiRNA biogenesis is a complex process that begins in the nucleus, with transcription, and finishes in the cytoplasm, with the maturation step [35,36]. In the nucleus, miRNAs are transcribed by RNA polymerase II into a precursor molecule, referred to as the primary transcript (pri-miRNA), which contains one or more sequences in a stem-loop structure [37,38]. The pri-miRNA undergoes two cleavages by Drosha, a class 2 RNase III-like enzyme, [39,40,41] in conjunction with DGCR8 (DiGeorge syndrome chromosomal region 8) [42,43,44]. This processing leads to the conversion of the pri-miRNA into a stem-loop intermediate structure with approximately 70 nucleotides called precursor (pre-) miRNA [22]. The pre-miRNA is exported to the cytoplasm by Exportin 5, being targeted by Dicer, a member of ribonuclease III (RNase III) family [19,45,46,47,48]. Dicer recognizes the pre-miRNA hairpin and cuts it into a smaller miRNA duplex, of approximately 22 nucleotides [49].

After being processed by Dicer, the mature miRNA is incorporated into the miRNA-induced silencing complex (miRISC), the final effector complex. The remaining strand exhibits less stable base pairing and associates with Argonaute (AGO) proteins, components of RISC that interact with miRNAs, as downstream effectors in the processes of translational repression through mRNA degradation or deadenylation [50,51,52,53].

Although several microRNAs with altered expression have been reported in AD, their function and mechanisms of regulation remain to be fully clarified. Accumulating evidence shows that microRNAs play a critical role in the pathogenesis of AD, being involved in the Aβ hypothesis (process regulation of Aβ production and clearance) and in the Tau hypothesis (imbalance between Tau phosphorylation and dephosphorylation in the formation of neurofibrillary tangles) [54]. Other microRNAs are involved in the regulation of neurogenesis, neuroinflammation, and insulin cascade, and may also contribute to the disease pathogenesis, as described in the next sections.

## 3. MiRNAs and AD Pathophysiology

**Figure 1:**
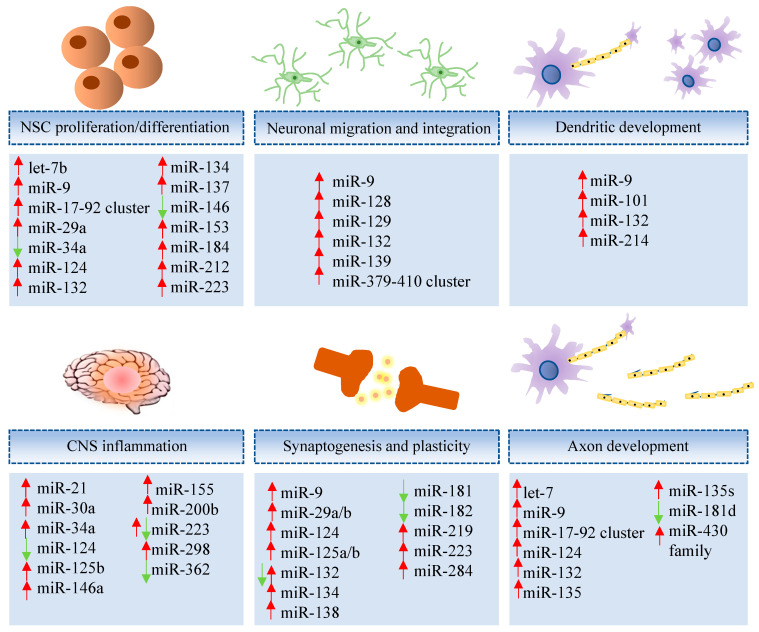
Representative miRNAs involved in neural development and function. Let-7b [58,59], miR-9 [58,59], miR-17-92 cluster [60], miR-29a [61], miR-34a [62], miR-124 [58], miR-132 [63], miR-134 [64], miR-137 [65], miR-146 [66], miR-153 [67], miR-184 [58], miR-212 [68], miR-223 [69], miR-9 [70], miR-128 [71], miR-129 [72], miR-132 [70], miR-139 [73], miR-379-410 cluster [74], miR-9 [75], miR-101 [76], miR-132 [77], miR-214 [78], miR-21 [79], miR-30a [80], miR-34a [81], miR-124 [81], miR-125b [82], miR-146a [83], miR-155 [81], miR-200b [79], miR-223 [57], miR-298 [79], miR-362 [79], miR-9 [84], miR-29a/b [57], miR-124 [84], miR-125a/b [64], miR-132 [64], miR-134 [64], miR-138 [58], miR-181 [85], miR-182 [86], miR-219 [87], miR-223 [57], miR-284 [88], let-7 [89,90], miR-9 [91], miR-17-92 cluster [60], miR-124 [84], miR-132 [92], miR-135 [93], miR-135s [93], miR-181d [94], miR-430 family [95].

MiRNAs play key roles in brain development, neuron differentiation, and synaptic plasticity [55], as illustrated in Figure 1. Moreover, deregulation of miRNAs is found in neurodegenerative diseases, including AD [56], involving processes of synaptogenesis, ion channel expression, inflammation, and neurodegeneration [57]. A review of microRNAs, from their role in AD, is found below and listed (Supplementary Tables S1 and S2).

### 3.1. MiRNAs in the Regulation of Neurogenesis

A decline of adult neurogenesis has been reported in brain samples obtained from AD patients when compared to controls [96]. Impaired neurogenesis seen in AD is probably the result of a process that compromises the ability for neural stem cell self-renewal, and deregulation of miRNAs is one of the mechanisms underlying age-related decline in neurogenesis, altering the normal function, self-renewal, differentiation, and survival of neural stem cells (NSCs).

Many are the reports of miRNAs that regulate NSC function, including miR-184 and miR-137 that, upon overexpression, increase NSC proliferation, and miR-124, miR-9, and lethal-7b (let-7b), which are associated with neural differentiation. MiR-223 was shown to induce neuronal morphogenesis in both human embryonic stem cells and adult mouse dentate gyrus NSCs [69]. The pro-neurogenic role of the miRNA-17-92 cluster was demonstrated in mice, as its ablation was associated with impaired adult hippocampal neurogenesis and loss of cognitive function [60].

MiR-9 is a brain-enriched miRNA that is expressed in the neurogenic areas of the embryonic and adult brain. During NSC differentiation, miR-9 expression is increased while TLX is decreased. This negative feedback loop could be regulating NSC proliferation and differentiation [58]. TLX also regulates transcription of miR-137 [65] and let-7 plays an important role in hippocampal neurogenesis, memory, and learning [59].

Notch pathway activation is required for normal NSC function and different miRNAs were linked to this regulation. MiR-184 and miR-34a are involved in NSCs’ maintenance, increasing self-renewal by regulation of Numbl, an inhibitor of Notch signaling. Knockdown of endogenous miR-34a in murine neural progenitor cells and NSCs resulted in increased expression of Numbl, NeuroD1, and Mash1 along with decreased *Notch1* expression [62,97]. A recent study in aged mice showed that miR-153 is downregulated in the hippocampus, and that miR-153 overexpression increased neurogenesis by interfering with the Notch signaling pathway [67].

The adult brain physiologically expresses miR-106b, miR-93, and miR-25 (members of the miR-106b~25 cluster), which target *Forkhead box O3* (*FoxO3*), a member of the forkhead transcription factors that orchestrates gene programs involved with insulin/insulin-like growth factor (IGF) signaling. *FoxO3* targeting by miR-106b increases neural progenitor cells and NSC proliferation, including neuronal differentiation in vitro, with implications for tissue homeostasis and aging [98].

Members of the let-7 family (let-7a and let-7b) have been identified as important regulators of NSCs’ fate [99,100]. Let-7 regulates *Hmga2*, a member of the high-mobility group A (HMGA) that is downregulated in NSCs with aging, reducing the proliferative capacity of both fetal and adult NSCs in mice [89]. Furthermore, it was demonstrated that miR-145 regulates the differentiation of NSCs by modulating the Sox2-Lin28/let-7 signaling pathway in vitro [90].

Some miRNAs are regulated in response to neuronal activity, including miR-134, which is modulated by myocyte enhancer factor 2 (MEF2) in response to membrane depolarization. MiR-132 is induced by cAMP response elements (CREB) activation and targets methyl CpG binding protein 2 (*MECP2*) [63]. *MECP2* also modulates miR-137 and miR-15a expressions [101,102]. MEF2 plays an important role in neuron survival [103] and deficiency in MECP2 protein leads to neuronal dysfunction [104].

In cortical neurogenesis, let-7b, miR-9, miR-124, and miR-134 are recruited to control the behavior of cortical progenitors. The involvement of miR-9 and miR-132 in neurite outgrowth has been described in embryonic mouse neocortex [70]. The absence of miR-132, along with miR-212, was shown to induce Tau aggregation and impair cognitive skills in an experimental model of AD. Additionally, high expression levels of these miRNAs were found in postmortem frontal cortex tissue from AD patients, suggesting possible involvement in the disease pathogenesis, as higher miR-132 and miR-212 levels were associated with improved AD patient cognitive scores [68].

### 3.2. MiRNAs and AD Neuroinflammation

A group of miRNAs displays neuroprotective functions and their deregulation plays critical roles in the pathogenesis of neurodegeneration. Some of these miRNAs are also involved in the regulation of inflammatory processes in neurological diseases, including miR-146a, one of the first miRNA found upregulated in AD patients’ brain tissues and in in vitro AD model [66]. Indeed, neuroinflammation plays a key role in AD pathogenesis [105,106] and increased secretion of proinflammatory cytokines leads to Tau hyperphosphorylation. These processes result from neuroinflammatory cascades, activation of microglia [106] and astrocytes, and their repercussions in glia-neuron interactions [107].

Brain inflammation appears to have a dual function, playing a neuroprotective role during an acute-phase response and protecting the brain from pathogens and neurotoxic agents and promoting tissue repair [108,109], but becomes detrimental when a chronic response is mounted [110]. Accumulated Aβ oligomers in AD trigger microglia activation through toll-like receptors, leading to chronic inflammation. Overexpression of miR-34a, which was described in AD human retinal tissues and in vitro model, leads to inhibition of Triggering receptor expressed on myeloid cells 2 (*TREM2*) expression in microglia, interfering with clearance of the oligomers [111,112]. Activation of the Nuclear factor-κB (NFκB) pathway in brain cells is associated with increased expression of inflammation-associated miRNAs as well as miR-155, miR-124, and miR-146a [81,113,114].

To note, miR-155 expression has been observed to be altered in brain tissue from patients with AD [115] as it displays a regulatory role in T-cell functions and, thus, AD progression [116]. Similarly, miR-124 is a potent negative regulator of Beta-Secretase 1 (BACE1) in the cellular AD phenotype and might be involved in the pathogenesis of AD [117]. MiR-146a and miR-181a are implicated in the mechanisms of AD progression as they are increased in patients with mild cognitive impairment who converted to AD. Their alterations are correlated to amyloid beta cerebrospinal fluid (CSF) concentration and associated to neuroimaging features [118].

Other miRNAs have been reported to be abnormally expressed and be closely correlated with the pathogenesis of AD, as follows. Let-7 family overexpression binds to TLR7 in AD-promoting neuroinflammation and compromises neuronal survival [119]. MiR-181 dysregulation in astrocytes may also contribute to the disease pathogenesis, since it has been linked to synaptic dysfunction [120]. In contrast, miR-21 plays a pivotal act in neuroinflammation as it can exert protective roles in AD, which might be dependent on Programmed Cell Death 4/Phosphoinositide 3-kinases/Protein kinase B/Glycogen synthase kinase 3 (PDCD4/PI3K/AKT/GSK-3β) signaling pathway in vitro [121].

### 3.3. MiRNAs, Insulin Resistance, and AD

Insulin resistance (IR) is central for the development of type 2 diabetes [122] and a growing body of evidence indicates that it is also a critical process for AD development, being associated with a failure in PI3K pathway activation [5,123]. Amyloid fibrils are polymers [124] produced by a precursor membrane protein called amyloid precursor protein (APP) [125]. APP cleavage by β- and Υ-secretases lead to the production of cytotoxic Aβ peptide with 40 or 42 amino acids (Aβ40-Aβ42) [126,127]. Aβ oligomers are associated with the development of neuronal insulin resistance (IR) [128].

It has been demonstrated that miR-29 family is the microglial modulators of the insulin-like growth factor-1 (IGF-1) and fractalkine ligand (CX3CL1) [129], supporting the hypothesis that impairment of IR and IGF-1 mediates cognitive impairment in AD [130]. MiR-375, miR-101, and miR-802 are involved in the regulation of gene expression in patients with type 2 diabetes [131] and are also present in mouse models of AD [132,133]. Increased levels of Aβ40-Aβ42 are usually seen in association with downregulation of miR-195 in AD [18]. At the early stages of AD in vivo, it has been reported that miR-132/212 are also downregulated [134].

Insulin receptors are found in CNS synapses in areas related to cognitive function and memory, such as the cerebral cortex and the hippocampus [135]. Insulin receptors can activate several different protein substrates, such as the insulin receptor substrate (IRS) family [136,137]. IRS-1 mediates glucose metabolism in AD [5,138], while IRS-2 mediates IR in type 2 diabetes [139]. However, it has been reported that miR-7 is overexpressed in the cerebellum and frontal cortex of AD patients, reducing the expression of IRS-2, impairing the insulin signaling pathway [140], and possibly modulating Aβ metabolism during disease progression. It has been reported that miR-126 is involved with micro- and macrovascular complications in type 2 diabetes [16], and this miRNA was found overexpressed in the cortex and hippocampus in a mouse model of AD [132,141]. MiR-126 regulates IRS-1 gene, reducing its expression, impairing the signaling pathway of PI3K-AKT, and, consequently, contributing to the development of IR.

Increased expression of miR-200b or -c diminishes secretion of the Aβ oligomers and, consequently, reduces IRS-1 phosphorylation in serine residues, which is associated with cognitive impairment and memory loss in AD [142,143,144]. Exacerbated IRS-1 phosphorylation on serine residues may also induce dephosphorylation of protein kinase B (AKT) by inhibiting pro-survival signaling pathways and activation of glycogen synthase kinase-3β (GSK-3β), a cellular pathway that leads to neuronal apoptosis [145]. GSK-3β, in turn, inhibits the induction of the autophagosome to the lysosome and stimulates the insertion of the autophagosome to the multivesicular body (MVB). MVB incorporates autophagosome, endosome containing some proteins, and exosomes containing proteins, DNA, mRNAs, and miRNAs, which later will be fused to the plasma membrane and are released into the extracellular medium in the form of extracellular vesicles (EVs) [146]. The EVs act in intercellular communication and are associated with the propagation of proteins such as APP and Tau that participate strongly in the pathophysiological process of AD. EVs are also related with the spread of several miRNAs that regulate gene expression at the post-transcriptional level, modulating many signaling pathways in AD [147].

Tumor necrosis factor (TNF-α) is highly expressed by microglia and astrocytes and activation of the TNF-α pathway contributes to IRS-1 phosphorylation, inhibiting PI3K pathway in AD [5]. Inflammatory responses mediated by microglial and astrocytes’ activation can be regulated by miR-21, which will contribute to avoid cognitive impairment and memory loss [148]. Nuclear factor of kappa light polypeptide gene enhancer in B-cells inhibitor alpha (IkBa), IκB kinase (IKK) and c-Jun N-terminal kinase (JNK) can be also activated in AD and lead to inhibition of IRS-1 phosphorylation in tyrosine residues with the concomitant increase of TNF-α [2]. Impaired insulin signaling pathway is linked to neuronal inflammation [4], synaptic loss, neurodegeneration, decreased neurogenesis, changes in behavior, cognitive impairment, and memory loss [149,150]. Conversely, memory enhancement was seen through the inhibition of miR-124, with concomitant downregulation of several genes [151].

### 3.4. MiRNAs and Aβ Production

Deregulation of miRNAs that target APP and β-site amyloid precursor protein cleaving enzyme 1 (BACE1) can lead to increased production and accumulation of Aβ peptides in the brain, with consequent synaptic failure, neurotoxicity, and dementia [152]. Different miRNAs have been reported to be involved in the regulation of Aβ expression, including miR-153 [153], miR-101 [154], miR-16 [155], miR-193b [156], miR-17, miR-20a, miR-106b, and miR-132, among others. [157].

Aβ peptides are fragments derived from APP, and miRNAs participate in the regulation of APP gene expression. MiR-101, miR-153, and miR-384 inhibit APP expression and, consequently, reduce the levels of Aβ peptide. These microRNAs were reported to be downregulated in vitro and AD patients [153,154,158]. MiR-29a, miR-29b-1, miR-29c, and miR-339-5p, which regulate BACE1, were found downregulated in AD, leading to BACE1 upregulation, with increased APP cleavage and Aβ accumulation in vitro, in vivo, and in the AD patients’ brain [61,159,160]. MiR-132 and miR-212 have also been found downregulated in AD patients and in 3xTg-AD mice, increasing the production of Aβ oligomers and senile plaque formation, while contributing to loss of cognitive function [161].

MiR-16, miR-101, miR-106a/b, miR-147, and miR-160a function as APP suppressors [133,162,163]. APP expression levels may influence the risk of AD [164]. Recently, it was described that miR-346 binds to the 5 ′ UTR of the APP mRNA, increasing its expression and translation, with consequent increase of Aβ production, leading to a cascade of redox stress and inflammation in vitro and in AD patients’ brain tissues [165]. These findings suggest that therapeutic modulation of the expression of miRNAs involved in the regulation of APP gene may impact Aβ accumulation.

One of the mechanisms associated with the accumulation of Aβ is the sequential proteolytic cleavage of full-length APP by BACE1. In vitro studies demonstrated that miR-29 is involved in the regulation of APP and BACE1 expression. In AD in vitro model, miR-29b negatively regulates BACE1 and subsequent Aβ peptides’ levels [166]. BACE1 is the aspartic-acid protease initiator for the formation of Aβ, and expression levels have been detected in AD brains. Several miRNAs have been reported to regulate BACE1 expression, including miR-339, miR-298, miR-328, miR-107, miR-200a, miR-124, miR-195, and miR-188. It was demonstrated that loss of miR-29 cluster increased BACE1 protein levels in AD [163]. Reduced miR-107 in AD patients’ brain neocortex samples negatively correlated with BACE1 [167].

MicroRNAs are also involved in the process of synaptic failure induced by Aβ. It was reported that inhibition of the expression of members of the miR-34 family could improve cognitive functions in the Aβ transgenic mice model and in cultured neurons exposed to Aβ [168,169]. MiR-188-5p was also demonstrated to restore the synaptic dysfunction and cognitive impairment evaluated in Aβ transgenic mice [170].

### 3.5. MiRNAs and Tau Hyperphosphorylation in AD

Hyperphosphorylated Tau protein is one of the hallmarks of AD and results from an imbalance between the activity of related kinases and phosphatases [171]. The accumulation of neurofibrillary tangles associated with tau hyperphosphorylation induces neuronal apoptosis, neurotoxicity, proteolysis, fibrillization, and neurodegeneration, impairing different brain functions, leading to AD [172] and cognitive deficits [173].

It was demonstrated in vivo that Tau expression is directly regulated by miR-137, miR-132, and miR-212. Interestingly, deletion of miR-132/212 in mice was associated with increased Tau phosphorylation and accumulation [134]. In brain tissues from AD patients, miR-219 was downregulated and associated with neurodegeneration, while in vitro assays confirmed Tau as its direct target [174].

Overexpression of miR-125b in hippocampal neurons is associated with changes in dendritic spine morphology and function [175]. In the context of AD, miR-125b is overexpressed, reducing the expression of dual-specific phosphatase 6 (DUSP6), protein phosphatase 1 catalytic subunit alpha isoform (PPP1CA), and B-cell lymphoma 2-like protein 2 (Bcl-W), resulting in the increase of p35, cdk5, and p44/42- mitogen-activated protein kinase (MAPK) signaling, hyperphosphorylation of Tau protein, and neurotoxicity, contributing to cognitive deficits in AD [173].

Increased miR-922 expression found in AD contributes to Tau phosphorylation, suppressing ubiquitin carboxy-terminal hydrolase L1 (UCHL1) in vitro [176], a protein involved in the maintenance of synaptic and cognitive function [177]. Similarly, overexpression of miR-146a in both in vitro (SH-SY5Y cells treated with Aβ1-42) and in a transgenic mouse model of AD suppressed the expression of rho-associated, coiled-coil-containing protein kinase 1 (ROCK1), reducing the phosphatase and tensin homologue (PTEN) phosphorylation, which contributes to GSK3 phosphorylation, finally promoting an exacerbation of phosphorylation of Tau protein. The accumulation of Tau in these models leads to the formation of forming neurofibrillary tangles, generating neuronal death, and favoring the progression of AD [178].

MiR-219, which showed reduced expression in post-mortem brain tissues obtained from AD patients, was associated with increased Tau gene expression and protein accumulation in the brain [174]. Likewise, reduced miR-106b expression in brain tissue samples from patients with AD was associated with increased expression of the proto-oncogene tyrosine protein kinase (*FYN*) gene, which has several biological functions from brain development to neuroinflammation, plasticity, and synaptic function and also contributes to Tau phosphorylation [179]. MiR-132 is also reduced in AD, being associated with increased expression of inositol 1,4,5-triphosphate 3 kinase B (ITPKB) and augmented Tau phosphorylation in the hippocampus of mice, favoring disease progression and cognitive dysfunction [180].

Another mechanism of epigenetic regulation of gene expression, histone deacetylation mediated by the histone deacetylases (HDACs), plays an important function in memory and synaptic plasticity [181]. The expression of HDACs is increased in AD precisely in the entorhinal cortex and in the hippocampus, contributing to increased cognitive deficits [182]. Interestingly, increased HDAC2 expression causes deacetylation of the hepatocyte nuclear factor 4a (HNF4A) transcriptional factor, inhibiting its expression and reducing the expression of miR-101b, which targets 5′ adenosine monophosphate-activated protein kinase (AMPK). Thus, AMPK is overexpressed, inducing tauopathy, promoting the progression of AD in a mouse model and comorbidities associated [183].

### 3.6. Circulating MiRNAs in AD

Stable extracellular miRNAs are present in body fluids and, therefore, circulating miRNAs have emerged as potential biomarkers for diagnosis, staging, and progress monitoring of various diseases. In AD, deregulated circulating miRNAs that are representative of the subjacent pathological process in the brain tissue could be utilized to support early diagnostics and to monitor disease progression. MiRNAs extracted from peripheral blood, serum, plasma, whole blood, or exosomes have been explored as biomarker candidates in AD [184] (Supplementary Table S3).

It is noteworthy that a circulating miRNA signature not necessarily correlates with brain tissue miRNA expression and underlying disease pathology. A systematic meta-analysis assessment was performed using 147 independent datasets across 107 publications. Deregulation of 32 miRNAs in the brain, CSF, and blood was reported. Five miRNAs exhibited significant differential expression in both brain and blood: The hsa-miR-181c-5p and hsa-miR-29c-3p (downregulated) and hsa-miR-125b-5p, hsa-miR-146a-5p, and hsa-miR-223-3p were upregulated in brain and downregulated in blood [185]. A review method was used to evaluate 8098 miRNAs in AD and controls from 26 studies. They found six downregulated miRNAs in AD patients: miR-107, miR-125b, miR-146a, miR-181c, miR-29b, and miR-342 [186,187].

Deregulated miRNAs in AD patients’ serum samples include miR-137, miR-181c, miR-9, and miR-29a/b [188]. The same miRNAs were also found downregulated in AD patients’ brain samples, showing a negative correlation with ceramide and Serine palmitoyltransferase, long chain base subunit 1/2 (SPTLC1/2) protein levels in the neocortex [189]. A genome-wide serum miRNA expression analysis identified six differentially expressed miRNAs in the serum of AD patients: miR-98-5p, miR-885-5p, miR-483-3p, miR-342-3p, miR-191-5p, and let-7d-5p [190]. Evaluation of 84 selected miRNAs in serum samples revealed downregulation of miR-125b, miR-23a, and miR-26b in AD patients [191]. Reduced levels of serum miR-125b was also found in AD. The same study reported miR-181c downregulation and miR-9 upregulation in serum samples of AD patients [192].

Interestingly, a study demonstrated that miR-384 regulates both APP and β-secretase expression. Downregulated miR-384 was found in CSF and serum compared with controls. In addition, the authors reported lower levels of this miRNA in blood samples from patients with AD dementia compared with mild cognitive impairment. [158].

Other studies evaluated miRNA expression profile in the CSF and exosome as a potential biomarker of AD. In the CSF, deregulation of several miRNAs including miR15b, miR-34a, miR-142, miR-146a, miR-125b, miR-545, and miR-29 family were found in AD patients compared to control subjects [193,194]. The expression levels of miR-193b, miR-135a, and miR-200b were associated with APP [156,195]. APP has an important function in the brain. However, the process responsible for the metabolism of APP to Aβ has not been well understood [196]. Six miRNAs were identified in plasma and CSF of AD patients as potential markers: miR-9, miR-29a, miR-29b, miR-34a, miR-125b, and miR-146a [197].

It was reported that circulating let-7d-5p, let-7g-5p, miR-15b-5p, miR-142-3p, miR-191-5p, miR-301a-3p, and miR-545-3p were upregulated in plasma samples from AD patients compared with normal controls [198]. Deregulation of miR-107 and miR-650 found in plasma samples of AD patients was associated with Aβ metabolism, contributing to pathological processes [199].

Finally, it was demonstrated that apolipoprotein E gene allele e4 is correlated with miR-146a levels in brain/plasma in mice and associated with increased AD risk [200]. Apolipoprotein E (ApoE) acts as a major lipid carrier in the brain and the isoform ApoE e4 has been identified as a strong AD genetic risk factor [201].

## 4. Physical Exercise

### 4.1. Beneficial Actions of Physical Exercise in AD

PE is currently recommended as a non-pharmacological measure for preventing cognitive decline [149,202,203]. Some studies have supported a role for PE in counteracting the pathophysiology determinants of AD and reducing the risk of memory loss in elderly, attenuating AD development [6,7]. Mechanisms reported involve enhanced brain plasticity [204], increased brain-derived neurotrophic factor (BDNF) [205], improved brain metabolism [206], and changes in the microbiome [207].

PE has been linked to improvement of cognitive status in AD patients [208] and attenuation of cognitive decline in aged individuals that express the APOE ε4 allele [209] and are predisposed to develop AD [210]. Glucose homeostasis is extremely important to synaptic plasticity and PE can contribute to manage it [211] and to inhibit oxidative stress [212].

Neuroprotective roles exerted by PE involve antioxidant and anti-inflammatory actions [9]. PE-induced reduction of neuroinflammation and improvements in memory were reported in AD experimental models [11,12,13]. Interestingly, transgenic AD mouse model submitted to an aerobic PE in the running wheel for three weeks showed reduced TNF-α and interleukin-1β (IL-1β) expression and decreased Aβ deposition in the brain [213]. Resistance training in 3xTg-AD mice also reduced the accumulation of Aβ and Tau protein, reduced expression of TNF-α and IL-1β, increased expression of IL-6, IL-10, Peroxisome proliferator-activated receptor gamma coactivator 1-alpha (PGC-1α), and fibroblast growth factor 21 (FGF-21), increased the expression of structural synaptic proteins, and improved cognitive function. These benefits may also be associated with AKT, GSK-3β, and JNK signaling pathways that were modulated by PE [214].

Converging with these findings, other studies report that PE mediates neuroprotection, increases antioxidant enzymes [14], decreases the expression of Aβ-42, Cox-2 and Caspase-3, cytochrome-c, and Bax, the phosphorylation of JNK, p38MAPK, and Tau, and exacerbated phosphorylation of extracellular signal-regulated kinase (ERK), PI3K, AKT, GSK-3α/β, NGF, BDNF, phospho-CREB, superoxide dismutase 1 (SOD-1), superoxide dismutase 2 (SOD-2), 70 kilodalton heat shock protein (HSP-70), and B-cell lymphoma 2 (BCL-2). Glucose, insulin, and corticosterone levels were also reduced after PE [15]. In an AD model, PE restored levels of non-protein thiols, preventing the accumulation of reactive oxygen species (ROS), increased SOD activity, prevented restored normal activity of glutathione reductase, glutathione peroxidase, and glutathione transferase, and suppressed TNF-α and IL-1β [215].

In healthy individuals, PE stimulates a better functioning of the PI3K pathway [123,216] through both insulin-dependent and -independent pathways [217,218]. PE also activates the β-oxidative pathway to degrade fat in order to produce energy [219]. Carnitine palmitoyl transferase (CPT) is a key enzyme complex in fat oxidation. Gene expression and the activity of the CPT complex is enhanced in muscle fibers of individuals who exercise [220]. CPT gene expression is modulated by peroxisome proliferator-activated receptor alpha (PPAR-α) and a co-activator (PGC-1α) [220]. PGC-1α leads to the production of fibronectin type III domain-containing 5 (FNDC5), which is cleaved at the C terminal to produce irisin [221].

Irisin is a myokine produced in the skeletal muscle during PE which correlated with increased IGF-1 levels in humans [222] and other growth factors in mice [223]. Irisin plays a role in synaptic plasticity, neurogenesis, cognitive function, and memory, but has unidentified receptors in the brain [139,149]. Irisin is supposed to be positively correlated to BDNF and IRS-1 activation in tyrosine residues in humans with AD, which may underline PE-mediated improvement of insulin resistance, neuroinflammation, and cognitive function in AD [149].

### 4.2. Modulation of MiRNAs by PE

**Figure 2:**
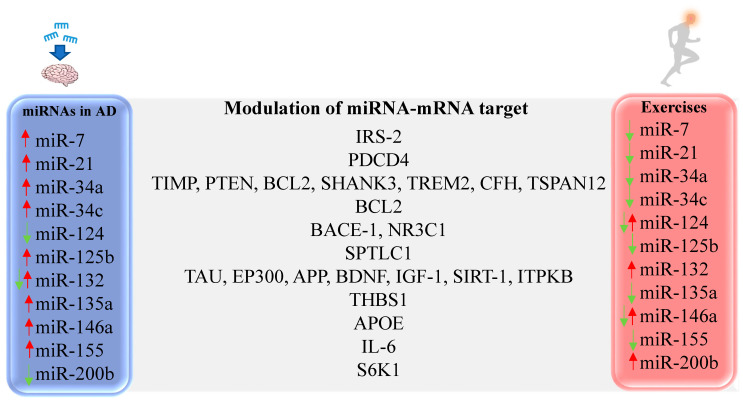
Representative miRNAs with altered expression in AD and differentially expressed after PE. IRS-2 [231,232], PDCD4 [121], TIMP [233], PTEN [233], BCL2 [234], SHANK3 [235], TREM2 [235], CFH [235], TSPAN12 [235], BCL2 [236], BACE-1 [237], NR3C1 [238], SPTLC1 [239], TAU [134], EP300 [240], APP [224], BDNF [227], IGF-1 [227], SIRT-1 [161], ITPKB [180], THBS1 [241], APOE [200], IL-6 [242,243], S6K1 [142].

Several studies have evaluated the role of PE in modulating miRNA expression both in the periphery and in the CNS, but the vast majority was performed in healthy individuals (Figure 2). An experimental study evaluated the effects of voluntary aerobic PE in the Senescence Accelerated Mouse-Prone 8 (SAMP8) mouse model of AD. While increased miR-132 was found in the hippocampus of untreated SAMP8 mice, PE was able to downregulate miRNA expression and reduce the accumulation of APP, inhibiting hippocampal degeneration and improving cognitive function [224]. In other preclinical studies, the expression of miR-132 was found reduced, which was also shown in AD patients and in vitro AD model [134,161,225,226]. Other studies have shown miR-132 upregulation in the brain after eight weeks of swimming training [227] and in trained human subjects [228,229,230]. Specificities of the evaluated model and time points may justify the discrepancies.

MiRNAs associated with fatty acid metabolism and biosynthesis signaling pathways, like miR-15b, miR-148b, miR-338, and miR-766, were also previously studied. MiR-15b is reduced in an in vitro AD model [244] and in brain samples of AD patients, leading to increased BACE-1 levels [245]. Chronic aerobic PE was able to increase hippocampal expression of miR-15b in SAMP8 mice [246], giving mechanistic support to the finding that PE reduces the expression of BACE-1 and decreases the accumulation of Aβ protein in the brain. Circulating miR-148b is increased in the blood of AD patients [187], but in healthy subjects, chronic aerobic PE reduced its expression [247]. MiR-338 circulating is downregulated in the plasma of AD patients [248], but PE increased expression of this miRNA in healthy subjects [228,247]. MiR-766 circulating is overexpressed in cerebral-spinal fluid of AD patients [249] but is reduced in plasma samples of healthy subjects after chronic PE [247].

MiR-124 was found downregulated in brain samples from AD patients [250] and in an in vitro AD model [237], in association with increased BACE-1 expression. Aerobic PE by chronic treadmill running [251] or chronic running wheel [252] increased miR-124 expression in the hippocampus and cerebral cortex, respectively, of healthy animals, which could contribute to reduce BACE-1 expression and Aβ accumulation.

Overexpression of miR-7 in AD plays an important role in Aβ metabolism by decreasing IRS-2 expression and suppressing insulin signaling pathway [140]. Meanwhile, after acute aerobic PE in a cycle ergometer, reduced expression of miR-7 was found in human serum samples [231]. Chronic swimming reduces IR in a type 2 diabetes mouse model by inducing miR-382 overexpression, which regulates resistin [253]. MiR-382 expression is reduced in brain samples of AD patients [250], bringing the hypothesis that IR may also be suppressed in AD by PE through regulation of miR-382.

Impaired insulin signaling and IR increases Aβ-mediated inflammatory process by modulating the IRS/PI3K/AKT/GSK3β signaling pathway [232], inducing neuroinflammation in AD [254,255]. In this context, increased activity of the transcription factor NFκB promotes elevation of miR-146a expression in brain tissues of AD patients [256], but acute resistance PE induced reduction of miR-146a expression [257] and the same result was obtained after three months of basketball training [258].

MiR-155 overexpression in brain samples of transgenic mouse model of AD was associated with activation of astrocytes and microglia, favoring increased expression of inflammatory mediators, including IL-6 [242]. In contrast, mice trained with endurance PE reduced expression of miR-155 and IL-6 expression was observed after six weeks of training [243].

MiR-137 is downregulated in both hippocampus and cerebral cortex of transgenic AD mice [259] and in serum samples of AD patients [188], contributing to Tau upregulation and phosphorylation. In contrast, hippocampal samples of mice that performed chronic aerobic PE with a running wheel showed elevation of miR-137 expression, improving memory in mice [260].

Overexpression of miR-125b is seen in AD and also favors Tau hyperphosphorylation [173]. However, it was observed that individuals who performed acute cycle ergometer exercise obtained reduction of miR-125b expression [228], a possible molecular mechanism to improve cognitive aspects and reduce neurotoxicity.

In the same perspective, miR-146a has been consistently found overexpressed in an in vitro AD model (SH-SY5Y cells treated with Aβ1-42), in a transgenic mouse model of AD [178], and in CSF, serum, and hippocampal samples of AD patients [261,262,263]. Individuals that underwent either acute resistance training or chronic basketball training reduced circulating levels of miR-146a [257,258]. This may be another signaling pathway for reducing Tau phosphorylation and that suppresses the formation of neurofibrillary tangles, minimizing neuronal death.

However, individuals who have done rowing training and after a marathon run, types of aerobic PE, had increased expression of miR-146a in serum and plasma samples, respectively [264,265], demonstrating that different types of training, the timing of the analysis, the protocol utilized, and type of sample can lead to divergent results regarding miRNAs’ expression patterns.

### 4.3. Overlaps Between MiRNA Signatures in AD and Physical Exercise

**Figure 3:**
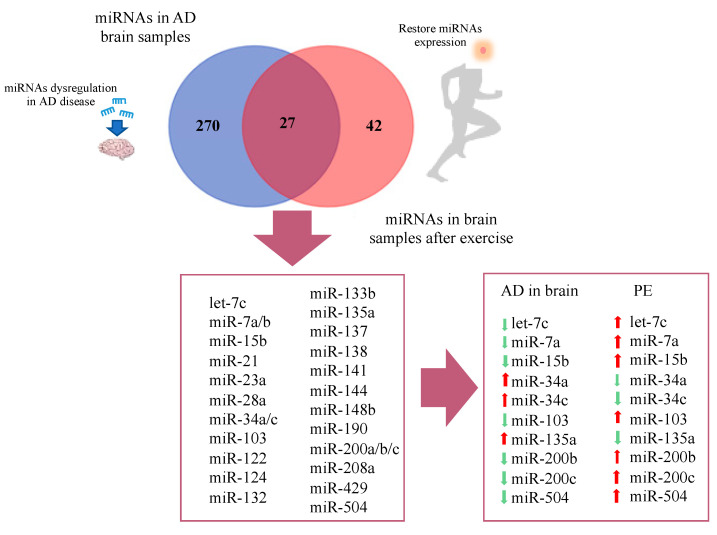
Venn diagram representing overlap of miRNAs in AD brain and restored miRNAs after physical exercise.

**Figure 4:**
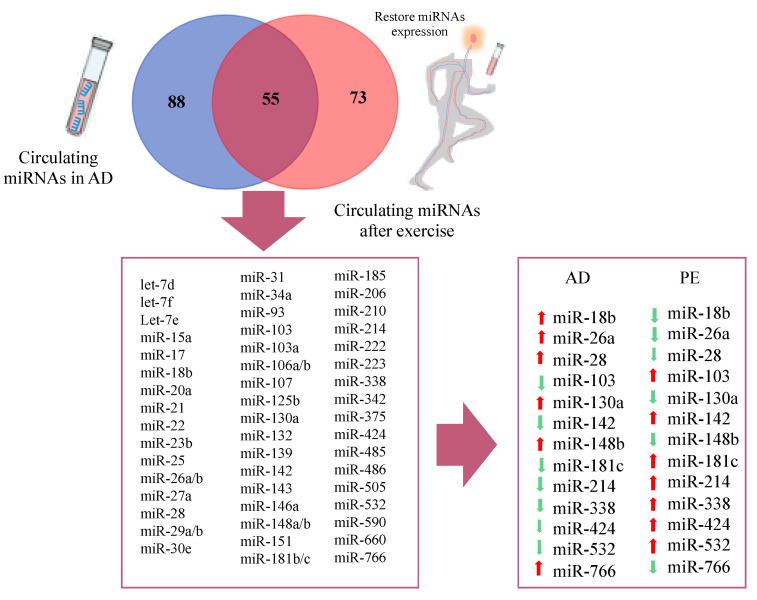
Venn diagram of circulating miRNAs in Alzheimer’s disease and restored miRNAs after physical exercise.

A number of studies have evaluated the impact of different modalities of PE in circulating and brain miRNA signatures (Supplementary Tables S4 and S5). Due to the lack of available results of PE-induced miRNA changes in AD subjects, we evaluated the commonalities between the lists of differentially expressed miRNAs reported in AD studies and in studies evaluating miRNA regulation by PE, mostly performed in healthy individuals. Overlaps were built, taking into account the lists of differentially expressed miRNAs, regardless of the pattern of expression (i.e., upregulated or downregulated) and Venn diagrams were constructed (Figure 3 and Figure 4).

Regarding studies evaluating brain tissue, a total of 270 differentially expressed miRNAs were reported exclusively in AD studies and 42 in PE studies, while 27 miRNAs were found overlapping AD and PE studies (Figure 3). The overlapping miRNAs were: let-7c, miR-7a, miR-7b, miR-15b, miR-21, miR-23a, miR-28a, miR-34a, miR-34c, miR-103, miR-122, miR-124, miR-132, miR-133b, miR-135a, miR-137, miR-138, miR-141, miR-144, miR-148b, miR-190, miR-200a, miR-200b, miR-200c, miR-208a, miR-429, and miR-504. Interestingly, from this list, 10 miRNAs showed opposite expression pattern comparing AD and PE studies, namely let-7c, miR-7a, miR-15b, miR-34a, miR-34c, miR-103, miR135a, miR-200b, miR-200c, and miR-504 (Figure 3).

**Figure 5:**
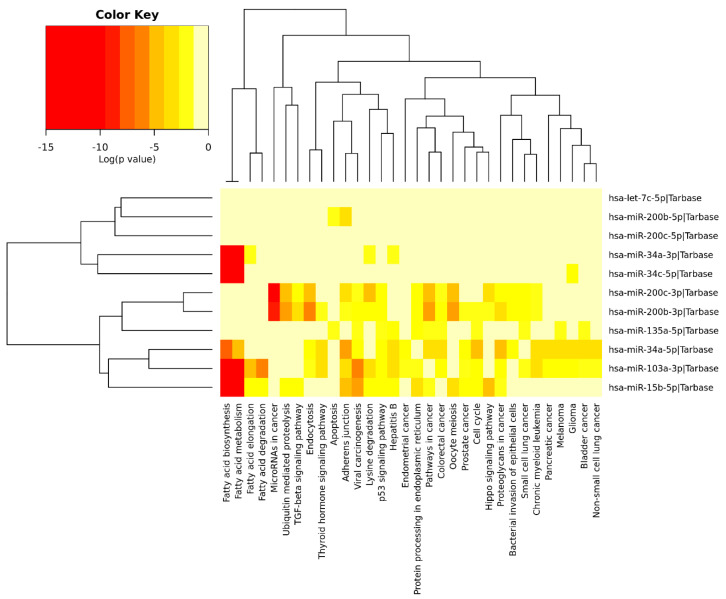
Heatmap clustering of miRNAs (brain samples) in Alzheimer’s disease vs. physical exercise and significant pathways’ interaction.

We performed an exploratory analysis of these 10 miRNAs by DIANA-miRPath v.3.0 integrated with TarBase v.7.0 and KEGG pathways. This database can determine the pathways that are regulated by multiple miRNAs using the hypergeometric test. Pathway enrichment analysis demonstrated association of these miRNAs with the following processes (excluding cancer and infectious disease-related processes): Fatty acid metabolism, ubiquitin-mediated proteolysis, protein processing in endoplasmic reticulum, endocytosis, cell cycle, adherens junctions, apoptosis, lysine degradation, p53 signaling pathway, hippo pathway, and transforming growth factor-β (TGF-β) pathway (Figure 5). Some of the identified pathways have been previously extensively studied in the context of AD. For instance, dysregulation of unsaturated fatty acid metabolism seems to be connected to AD severity [266], and PE induces changes in fatty acid metabolism, as well as modifying the expression of inflammatory adipokines and increased lipolysis in white adipose tissue [267], including elevating the expression of enzymes associated with the metabolism of polyunsaturated fatty acids [268].

**Figure 6:**
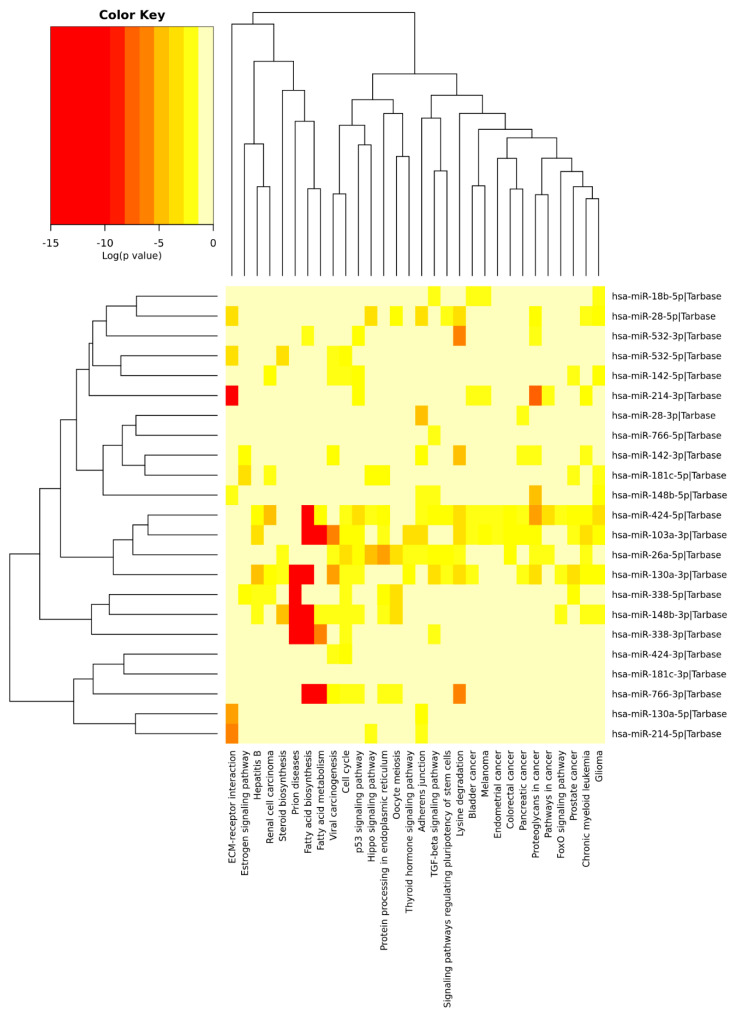
Heatmap clustering of circulating miRNAs in Alzheimers’ disease (AD) vs. physical exercise (PE) and significant pathways’ interaction.

Similar analysis was performed for circulating miRNAs reported in AD studies and PE studies. A total of 88 differentially expressed circulating miRNAs were reported exclusively in AD studies and 73 in PE studies, while 55 miRNAs were found overlapping AD and PE studies (Figure 4), from which 13 showed opposite expression patterns: miR-18b, miR-26a, miR-28, miR-103, miR-130a, miR-142, miR-148b, miR-181c, miR-214, miR-338, miR-424, miR-532, and miR-766. Although a different list of miRNAs was found when compared to brain miRNAs, similar signaling pathways were found (Figure 6). Interestingly, miR-103 was found in both circulating and brain miRNA analyses. In a recent study, miR-103 was shown to target A disintegrin and metalloproteinase domain-containing protein 10 (ADAM10), which is the most important enzyme with α-secretase activity involved in the processing of APP and the formation of amyloid plaques [269], Supplementary Tables S1–S5 ([233,234,235,236,238,239,240,241,270,271,272,273,274,275,276,277,278,279,280,281,282,283,284,285,286,287,288,289,290,291,292,293,294,295,296,297,298,299,300,301,302,303,304,305,306,307,308,309,310,311,312,313,314,315,316,317,318,319,320,321,322,323,324,325,326,327,328,329,330,331,332,333,334,335,336,337,338,339,340,341,342,343,344,345,346,347,348,349,350,351,352,353,354,355,356,357,358,359,360,361,362,363,364,365,366,367,368,369,370,371,372,373,374,375,376,377,378,379,380,381,382]).

## 5. Conclusions

MiRNA deregulation participates in different aspects of AD pathogenesis. Although PE is a non-pharmacological measure recommended for AD patients, the mechanisms involved in the associated benefits are not fully understood. Current evidence is insufficient to prove but can provide significant biological plausibility to support a possible role for PE in counteracting some of the deregulated miRNAs found in AD, and to generate hypotheses regarding which integrated pathway clusters may be involved. PE may act on restoring a miRNA profile that supports normal NSC function, brain metabolism, and inflammatory status, among others. Further studies, however, are required to advance this knowledge. Finally, the study of miRNAs involved in the beneficial role of PE in AD may also contribute to identify biomarkers and to develop new drugs and therapies for the disease in the future.

## Supplementary Materials

Supplementary materials can be found at https://www.mdpi.com/1422-0067/21/14/4977/s1.
