# Supplementary material for: Modulation of MicroRNAs as a Potential Molecular Mechanism Involved in the Beneficial Actions of Physical Exercise in Alzheimer Disease
Source: Int J Mol Sci. 2020 Jul 14;21(14):4977. doi: 10.3390/ijms21144977 (PMC7403962; doi:10.3390/ijms21144977)
Supplement: Supplementary file 1 [file ijms-21-04977-s001.pdf]

Supplementary Tables

Supplementary Table S1. Preclinical studies evaluating miRNA deregulation in brain associated with AD.

	MicroRNAs	Targets	Source	Models	Reference
⬆	miR-146a	CFH	Hippocampal and superior temporal lobe neocortical tissues samples	AD Human Brain Cells	[66]
⬇	miR-107	BACE1	Human cerebral cortex samples	AD patients and cell culture	[270]
⬇	miR-298, miR-328	BACE1	Hippocampus samples	APP/PS1 mice	[271]
⬆	miR-34a	BCL2	Cerebral cortex samples	Transgenic mouse model of AD	[234]
⬇	miR-17-5p, miR-20a, miR-106b	APP	Temporal cortex and cerebellum samples	AD human brain; HeLa cells; Neuro2A and human SK-N-SH cells	[163]
⬇	miR-101	APP	Hippocampal neurons samples	Hippocampal neurons of wistar rats	[133]
⬇	miR-9, miR-20b, miR-21, miR-148b, miR-181c, miR-361, miR-409, let-7i	MAPK, MTOR, ERBB	Hippocampal neurons samples	Primary hippocampal neuron cells	[272]
⬆<br>⬇	miR-106b<br>miR-106b	TβR-II	Cerebral cortex samples	APPswe/PSΔE 9 mice	[273]

⬇	miR-103, miR-107	COFILIN	Brain tissue samples	Tg19959 AD mice model	[274]
⬆	miR-146a		Brain tissue samples	Transgenic animals Tg2576	[275]
⬆	miR-34c	BCL2	Hippocampus samples	Mouse model of AD	[236]
⬇	miR-101	APP	HeLa and Human neuroblastoma cells	Human neuroblastoma cells	[154]
⬇	miR-153	APP	Human fetal brain cells and Frontal cortex samples	Primary human brain cultures and AD patients	[153]
⬇	miR-195	BACE1	Hippocampus samples	SAMP8 AD mice model	[276]
⬇	miR-124	BACE1	Brain cortex samples	Cellular AD model	[237]
⬇	miR-16	APP	Hippocampus samples	SAMP8 AD animal model	[162]
⬆	miR-206	BDNF	Brains samples of Tg2576 Temporal cortex samples of human AD patients	Tg2576 AD transgenic mice and AD patients	[277]
⬇	miR-153	APP, APLP2	Cerebral cortex samples	APPswe/PSΔE9 AD murine model	[278]
⬆	miR-30a-5p	BDNF	Primary cortical neuron samples	Ab25–35 AD neuron model	[279]

↑	miR-144, miR-451	ADAM10	SH-SY5Y cells samples	Ab42 SH-SY5Y AD model	[280]
↓	miR-132, miR-212	P300, PTEN, FOXO3a	Temporal cortex samples	AD patients and APP PS1 mice	[281]
↑	miR-98, let-7d, let7e	IGF-1	Hippocampus samples	APP/PS1 AD mice model and HEK293 cells	[282]
↓<br>↑	miR-1,miR-20b, miR-26b, miR-27a, miR-27b, miR-32, miR-107, miR-126-5p, miR-128a, miR-129-3p, miR-133a, miR-133b, miR-137, miR-141, miR-142-3p, miR-142-5p, miR-147, miR-148a, miR-148b, miR-150, miR-151-3p, miR-152, miR-181a, miR-181c, miR-202-3p, miR-219, miR-223, miR-301b, miR-325, miR-330, miR-337-3p, miR-338-3p, miR-339-5p, miR-342-5p, miR-345-3p, miR-375, miR-381, miR-429,miR-449a, miR-455, miR-489, miR-547, miR-582-5p, miR-666-5p, miR-770-3p<br>miR-99a, miR-103, miR-122, miR-124, miR-125a-3p, miR-125b-5p, miR-126-5p, miR-139-3p, miR-146a, miR-146b, miR-148a, miR-152, miR-193, miR-224, miR-298, miR-325, miR-335-5p, miR-337-5p, miR-340-5p, miR-344, miR-345-5p, miR-467c, miR-467e, miR-504, miR-547, miR-582-3p		Hippocampus samples	3xTgAD mice	[132]
↑	miR-26b	Cyclin E1, p27, Kip1	Brain tissue and neurons samples	AD patients and primary cortical neurons	[283]
↑	miR-20a, miR-130b, miR-193b, miR-296, miR-329	MAK	Hippocampus samples	SAMP8 and SAMP10 mouse model	[284]
↓	miR-188	BACE1	Brain tissues samples	AD patients and APP transgenic mice	[285]
↑	miR-206	BDNF	Hippocampal tissue, CSF and plasma samples	APP/PS1 transgenic mice	[286]
↑	miR-28a-5p, miR-98-5p, miR-148b-3p	HDAC6 Sirt1	Hippocampal tissue samples	SAMP8 mice	[246]

↑	miR-155	SOCS-1	Brain tissue samples	Transgenic mouse model	[242]
↑	miR-125b	DUSP6, PPP1CA, Bcl-W	Brain tissue and neurons samples	AD patients, primary hippocampal and cortical neurons	[173]
↑<br>↓	miR-7b-5p, miR-99b-5p, miR-100-5p, miR-331-3p, miR-434-3p<br>miR-7a-5p, miR-7b-5p, miR-99b-5p, miR-100-5p, miR-409-5p, miR-501-3p		Brain tissue samples	APPswe/PS1DE 9 AD Mouse Model	[287]
↓	miR-384	APP	Hippocampal samples form the animals and Plasma and serum samples form the humans	Mouse model of AD and patients with AD	[158]
↓	miR-193b	APP	Hippocampus samples of mouse model and cerebral spinal fluid of AD patients	Transgenic mice and AD patients	[156]
↑	miR-126	IRS-1, SPRED-1, DLK1	Cortex and hippocampus samples	Tg6799 transgenic mice	[141]
↑	miR-181	c-FOS, SIRT-1	Hippocampus samples	3xTg-AD mice	[288]
↓	miR-339-5p	BACE1	Human fetal brain cells and Frontal cortex samples	Primary human brain cultures and AD patients	[159]
↑<br>↓	miR- 8, miR-13b, miR-277, miR-279, miR-981, miR-995, miR-998, miR-1017<br>let-7, miR-1, miR-9a, miR-184, miR-193, miR-263b, miR-276a, miR-285, miR-289		Brain tissue samples	AD Drosophila model	[289]

↑	miR-138	RARA	N2a/APP and HEK293/tau cells samples	N2a/APP and HEK293/tau cells model	[290]
↓	miR-29c	BACE1	Brain tissue samples	AD patients and SH-SY5Y cells	[291]
↑	miR-29c	NAV3	Hippocampus samples	APPswe/PSΔE9 AD mouse model	[292]
↓	miR-222	p27	Brain tissue samples	APPswe/PSΔE9 AD mouse model	[293]
↓	miR-132, miR-212	TAU	Brain samples	miR-132/212 knockout mice and Neuro2a cells	[134]
↑	miR-135a	THBS1	Cerebral samples	AppTg mice and U373MG cells	[241]
↓	miR-124	Delta, Notch	Brain tissue samples	Drosophila AD model	[294]
↓	miR-132		Brain and neurons samples	AD patients and AD cell model	[225]
↑	miR-26b	IGF-1	Brain tissue samples	APP/PS1 Transgenic mouse model of AD	[295]
↑	miR-146a	ROCK1	Neural SH-SY5Y cells	APP-SH-SY5Y AD model	[178]
↓	miR-132, miR-212	SIRT-1	Hippocampus samples	Neuro2a cells, 3xTg-AD [161]mice model and	[161]

				AD patients	
↓	miR-181c	CRMP2	Hippocampus samples	SAMP8 mice	[296]
↓	miR-214-3p	ATG12	Cerebrospinal fluid and hippocampus samples	SAMP8 mice model and AD patients	[297]
↓	miR-125b	p53, BAK1	Primary cortical neurons samples	AD cell model	[298]
↑	miR-34a	SYT1, HCN1, NR2A, GluR1, NDUFC2, SDHC, UQCRB, UQCRQ, COX10, H6PD, PFK1, PFK2	Brain tissue samples	AD patients and 3xTg-AD mouse model	[299]
↓	miR-146a	IRAK1	Brain and plasma samples	AD mice model	[200]
↓	miR-9	CAMKK2	Primary cortical neurons	AD cell model	[300]
↓	miR-132	ITPKB	Hippocampus samples	AD patients and AD mice model	[180]
↑	miR-98-5p	SNX6	SK-N-SH and SH-SY5Y cells samples	SK-N-SH, SH-SY5Y and HEK293 cells	[301]

↑	miR-613	BDNF	Hippocampus, CSF and serum samples	AD patients and APP/PS1 AD mice model	[302]
↑<br>↓	miR-195<br>miR-15b	MFN2	Hippocampus samples	SAMP8 mice model	[303]
↓	miR-188-5p	Nrp-2	Hippocampus samples	AD patients and 5XFAD mice	[170]
↓	miR-132, miR-212	NOS1	Brain tissue samples	AD patients, AD mouse model and cell culture	[226]
↑	miR-125b	FOXQ1	Postmortem brain samples	Neurons cells culture	[172]
↑	miR-139	CB2	Hippocampus samples	SAMP8 mices and hippocampal cell culture	[304]
↑	miR-146a, miR-155	IRAK-1, TRAF6	Hippocampus samples	APP/PS1 AD mice model and AD patients	[263]
↓	miR-101b	AMPK	Brain tissue samples	AD mouse model and cell culture	[183]
↑	miR-106b		Brain tissue samples	AD patients APP/PS1 AD mice model and SH-SY5Y cell culture	[305]
↑	let-7a	PI3K, p-AKT, p-mTOR	PC12 and SK-N-SH sample	PC12 and SK-N-SH AD model	[306]
↑	miR-376c-3p, miR-342-5p, miR-878-3p, miR-342-3p, miR-1940, miR-5624-5p, miR-3074-5p, miR-450a-5p, miR-2136, miR-3058-3p, miR-342-5p, miR-344i, let-7f-5p,		Brain samples	APP/PS1 AD mice model	[307]

	miR-3059-3p, miR-218-2-3p, miR-539-3p, miR-3967, miR-431-5p, miR-1961, miR-10a-5p, miR-450a-5p, miR-742-5p, miR-2136, miR-301b-3p, miR-302b-5p, let-7d-3p, miR-211-5p, miR-98-5p, miR-342-3p, miR-292-5p, miR-1251-5p, miR-743a-5p, miR-1a-3p, miR-133a-3p, miR-652-5p, let-7f-5p, miR-215-5p, miR-133b-3p, miR-34a-5p, miR-881-5p, miR-135a-2-3p, miR-135b-5p, miR-34c-3p, miR-431-5p, miR-187-3p, miR-376c-3p, miR-712-3p, miR-342-3p, miR-5626-5p, miR-3085-3p, miR-128-2-5p, miR-291a-3p, miR-3106-3p, miR-331-5p<br><br>⬇ miR-499-3p, miR-3070a-5, miR-3070b-5p, miR-291a-3p, miR-491-3p, miR-25-5p, miR-3073a-3p, miR-491-3p, miR-299a-3p, miR-1964-5p, miR-33-3p, miR-879-3p, miR-299a-5p, miR-299b-5p				
⬇	miR-15b	BACE1	Brain samples	AD patients and SH-SY5Y cell culture	[245]
⬆	miR-200a-3p	SIRT1	Hippocampus samples	APPswe/PS delta E9 AD mice model and PC12 cells AD model	[308]
⬇	miR-124	BACE1	Frontal cortices samples	AD patients, SH-SY5Y and HEK293 cell culture	[117]
⬆	miR-124	PTPN1	Hippocampus sample	Tg2576 mice	[309]
⬇	miR-144-5p, miR-221, miR-374	ADAM10	Blood samples and SH-SY5Y samples	AD patients and SH-SY5Y cell culture	[310]
⬇	miR-873-5p	HMOX1	Brain tissues and cells samples	SAMP8 AD mice model and PC12 cells AD model	[311]
⬆	miR-125b	SPHK1	APPSwe/Δ9 cells and CSF sanmples	AD patients and APPSwe/Δ9 cells	[312]

⬇	miR-15b	BACE1, NFKB	SH-SY5Y cells samples	SH-SY5Y cells AD model	[244]
⬇	miR-16	BACE1	Frontal brain cortices and cells samples	AD patients and PC12 cells AD model	[155]
⬆	miR-10a		Brain and cell samples	AD rats model	[313]
⬆<br>⬇	miR-1233-3p, miR-3622-3p, miR-6845-3p<br>miR-4487		SH-SY5Y cells samples	SH-SY5Y cells AD model	[314]
⬇	miR-137	TAU	Hippocampus and cerebral cortex samples	Double-transgenic AD mice	[259]
⬇	miR-219-5p	TTBK1, GSK-3b	Brain tissues and SH-SY5Y cells samples	AD patients, APP/PS1 AD mice model and SH-SY5Y cells AD model	[315]
⬇	let-7f-5p	Caspase3	MSCs samples	MSCs treated with Aβ25-35	[316]
⬆	miR-132	APP	Hippocampus, cortex and	SAMP8 mice model	[224]
⬆	miR-21	PDCD4	SH-SY5Y cells	AD model SH-SY5Y	[121]
⬇	miR-330	VAV1	Cerebral cortex samples	AD mice model	[317]
⬇	miR-101a	APP	SH-SY5Y cells samples	AD cell model	[318]
⬇	miR-200b, miR-200c	S6K1	Brain cortex samples of AD mice models	Neuronal cells culture and mice model of AD	[142]
⬆<br>⬇	let-7e-5p, miR-128-3p, miR-191<br>let-7c-5p, let-7e-5p, miR-26b-5p, miR-29a-3p, miR-146a-5p, miR-181a-5p	HMG20B NUP160	Hippocampal samples	SAMP8 AD mice model	[319]

		SOX6 BDNF			
↑	miR-30b	EphB2, SIRT1, GluA2	Hippocampal tissues and cells	AD patients, NG108-15 and HEK 293/293T cells	[320]
↓	miR-133b	EGFR	SH-SY5Y cells samples	AD patients and SH-SY5Y cells AD mode	[321]
↑	miR-134-5p	CREB-1, BDNF	Hippocampus samples	Aβ- treated rat hippocampus cells	[322]
↑	miR-128	PPARγ	Cerebral cortex samples	AD mice model	[323]
↓	miR-98	HEY2	Hippocampal samples	AD mice model	[324]
↓	miR-200a-3p	BACE1	Hippocampus of APP/PS1 and SAMP8 mice. Blood plasma samples	APP/PS1, SAMP8 mice and AD patients	[325]

Supplementary Table S2. Brain miRNA deregulation reported by clinical studies in AD.

MicroRNAs	Targets	Source	Reference
↑ miR-9, miR-128	SPTLC1, PPARγ	Hippocampus	[326]
↑ miR-26a, miR-27a, miR-27b, miR-30c, miR-30e, miR-34a, miR-92, miR-100, miR-125b, miR-148a, miR-200c, miR-381, miR-422a, miR-423<br><br>↓ miR-9, miR-26a, miR-29, miR-30c, miR-132, miR-145, miR-146b, miR-200c, miR-210, miR-212, miR-425	TIMP-3 PTEN	Hippocampus, medial frontal gyrus, and cerebellum	[233]
↓ miR-29a, miR-29b-1	BACE1	Anterior temporal cortex or cerebellum	[61]
↑ miR-9, miR-125b, miR-146a	SPTLC1, FOXQ1	Temporal lobe neocortex	[239]
↓ miR-29a	NAV3	Frontal cortex	[327]
↑ miR-146a	IRAK-1	Temporal lobe neocortex	[256]
↓ miR-107	BACE1	Temporal cortex	[167]
↑ miR-519e, miR-574-5p, miR-498, miR-518a-5p/miR-527, miR-525-5p, miR-300, miR-576-3p, miR-583, miR-146b-3p, miR-490-3p, miR-549, miR-516a-5p, miR-510, miR-184, miR-516b, miR-298, miR-214, miR-198, miR-451, miR-144, miR-424, let-7e, miR-509-5p, miR-574-3p, miR-576-5p, miR-302e, miR-220b, miR-208a, miR-215<br><br>↓ miR-485-3p, miR-381, miR-124, miR-34a, miR-129-5p, miR-29a, miR-143, miR-136, miR-145, miR-138, miR-129-3p, miR-128, miR-379, miR-299-5p, miR-218, miR-149, miR-135a, miR-7, miR-126, miR-411, miR-335, miR-9, miR-378, miR-488, miR-432, miR-127-5p, miR-127-3p, miR-491-5p, miR-376c, miR-377, miR-95, miR-222, miR-29b, miR-329, miR-495, miR-551b, miR-195, miR-125b, miR-30b, miR-221, miR-139-5p, miR-487a, miR-487b, miR-107, miR-146b-5p, miR-29c, miR-30a, miR-582-5p, miR-103, miR-342-3p, miR-331-3p, miR-30c, miR-30d, miR-382, miR-22, miR-125a-	N/A	Superior and middle temporal gyri	[250]

	5p, miR-491-3p, miR-423-5p, miR-34b, miR-422a, miR-34c-5p, miR-584, miR-219-5p, miR-338-3p, miR-219-2-3p, miR-338-5p, miR-181a, miR-181b, let-7b, miR-151-3p, miR-197, miR-19a, miR-20a, miR-17, miR-106a, miR-32, miR-340, miR-19b, miR-21, miR-151-5p, miR-194, let-7c, miR-330-3p, miR-27b, miR-93, miR-15a, miR-339-5p, miR-193b, miR-106b, miR-16, miR-23b, miR-15b, miR-320d, miR-320b, miR-320c, miR-320a, miR-557, miR-33a, let-7a, miR-374b, miR-140-3p, miR-374a, miR-24, miR-140-5p, miR-26a, miR-513a-5p, miR-212, miR-142-5p, miR-142-3p, miR-26b, miR-520d-5p, miR-193a-3p, miR-92b, miR-330-5p, miR-186, let-7f, miR-223, miR-412, miR-185, miR-148b, miR-101, miR-99b, miR-27a, miR-589, let-7i, miR-361-3p, miR-361-5p, miR-423-3p, miR-190, miR-301a, miR-365, miR-23a, miR-363, miR-326, miR-425, miR-191, miR-519d, let-7g, miR-98, miR-99a, miR-30e			
↓	miR-9, miR-15, miR-29a, miR-29b-1, miR-124, miR-137, miR-181c	SPTLC1<br>SPTLC2	Neocortical Brain samples	[189]
↑<br>↓	miR-15a<br>miR-370		Cerebellum, hippocampus, CSF and plasma	[328]
↑	miR-125b	DUSP6, PPP1CA, Bcl-W	Frontal cortex	[173]
↑<br>↓	miR-16, miR-34c, miR-146a<br>miR-16, miR-107, miR128a, miR-146a		Hippocampus	[329]
↓	miR-219	TAU	Frontal cortex	[174]
↓	miR-512, miR-765, miR-1181, miR-1292	cFLIP, MCL1	Brain tissue samples	[330]
↑	miR-603	LRPAP1	Hippocampus	[331]
↓	miR-106b	FYN	Temporal cortex	[179]
↓	miR-15b, miR-16, miR-103, miR-107, miR-195	CDK5R1, p35	Hippocampus and	[332]

			temporal cortex	
↓	miR-132-3p, miR-212-3p		Human brain tissue	[333]
↑	miR-455-3p, miR-3613-3p, miR-4674, miR-6722	THBS1, COL3A1, RUXN1, TP73, EXT1, CDKN2A, PSME3	Frontal cortex	[334]
↓	miR-129, miR-132	EP300	Dorsolateral prefrontal cortex	[240]
↑	miR-34a, miR146a	SHANK3, TREM2, CFH, TSPAN12	Hippocamp us	[235]
↑	miR-140-5p	ADAM10	Hippocamp us	[335]
↑	miR-346	APP	Frontal cortex	[165]
↑	miR-455	APP, NGF	Frontal cortex	[336]

Supplementary Table S3. Circulating miRNA deregulation in AD: preclinical and clinical studies.

	MicroRNAs	Source	Models	Reference
↑	miR-342-5p	Serum	AD transgenic mouse model	[337]
↑	miR-206	CSF and plasma	APP/PS1 transgenic mice	[286]
↓	miR-193b	CSF	Transgenic mice and AD patients	[156]
↓	miR-135a, miR-200b	CSF	Transgenic mice and AD patients	[195]
↑<br>↓	miR-346<br>miR-132, miR-138, miR-139, miR-146a, miR-146b, miR-22, miR-24, miR-29a, miR-29c	Plasma	3xTg-AD model	[338]
↓	miR-146a	Plasma	AD mice model	[200]
↑	miR-613	CSF and serum	AD patients and mice model	[302]
↑	miR-200c	Plasma samples	AD patients and APP/PS1 AD mice model	[339]
↓	miR-214-3p	CSF	SAMP8 mice model and AD patients	[297]
↓	miR-135b	Peripheral blood	AD patients and SAMP8 mice	[340]
↑<br>↓	miR-28-3p<br>miR-9, miR-125b, miR-191-5p	Serum	APP/PS1 AD mice model	[341]
↓	miR-200a-3p	Plasma	SAMP8 mice and AD patients	[342]
↑	miR-34a, miR-181b	Blood	AD patients	[343]
↓	miR-137, miR-181c, miR-9, miR-29a, miR-29b	Serum	AD patients	[188]
↑	miR-9, miR-125b, miR-146a, miR-155	CSF	AD patients	[261]
↑<br>↓	miR-323b-5p, miR-563, miR-600, miR-1274a, miR-1975<br>let-7d-5p, let-7g-5p, miR-15b-5p, miR-142-3p, miR-191-5p, miR-301a-3p, miR-545-3p	Plasma	AD patients	[198]
↑	miR-15a	CSF and plasma	AD patients	[328]
↑<br>↓	miR-112, miR-161, let-7d-3p, miR-5010-3p, miR-26a-5p, miR-1285-5p, miR-151a-3p<br>miR-103a-3p, miR-107, miR-532-5p, miR26b-5p, let-7f-5p	Blood	AD patients	[344]
↓	miR-27a-3p	CSF	AD patients	[345]

↓ miR-125b, miR-23a, miR-26b	Serum and CSF	AD patients	[191]
↑ miR-9<br>↓ miR-125b, miR-181c	Serum	AD patients	[192]
↑ miR-3158-3p, miR-27a-3p, miR-26b-3p, miR-151b<br>↓ miR-36, miR-98-5p, miR-885-5p, miR-485-5p, miR-483-3p, miR-342-3p, miR-30e-5p, miR-191-5p, let-7g-5p, let-7d-5p	Serum	AD patients	[190]
↑ miR-361-5p, miR-30e-5p, miR-93-5p, miR-15a-5p, miR-143-3p, miR-335-5p, miR-106b-5p, miR-101–3p, miR-425-5p, miR-106a-5p, miR-18b-5p, miR-3065-5p, miR-20a-5p, miR-582-5p<br>↓ miR-1306-5p, miR-342-3p, miR-15b-3p	Serum exosomes	AD patients	[346]
↑ miR-34c	Plasma and PBMC	AD patients	[347]
↓ miR-146a	CSF	AD patients	[329]
↑ miR-29a, miR-29b<br>↓ miR-34a, miR-125b, miR-146a	CSF and plasma	AD patients	[197]
↑ miR-128	Blood and PBMCs	AD patients	[348]
↓ miR-384	Plasma and serum	AD patients	[158]
↓ miR-146a-5p	Blood	AD patients	[349]
↑ miR-590-5p, miR-486-5p<br>↓ miR-129-3p, miR-139- 3p, miR-181-5p, miR-210, miR-223-5p, miR-374b, miR-519-3p, let-7a, miR-424-3p, miR-532-3p, miR-758	CSF	AD patients	[350]
↑ miR-130a-3p, miR-339-5p, miR-425-5p, miR-3607-3p, miR-4297<br>↓ miR-25-5p, miR-639, miR-5000-5p, miR-5699	PBMC	AD patients	[351]
↓ miR-29c, miR-136-3p, miR-16-2, miR-331-5p, miR-132-5p, miR-151, miR-485-5p	CSF	AD patients	[352]
↓ miR-31, miR-93, miR-143, miR-146a	Serum	AD patients	[184]
↑ miR-548at-5p, miR-138-5p, miR-5001-3p, miR-659-5p	Plasma exosomes	AD patients	[248]

↓ miR-185-5p, miR-342-3p, miR-141-3p, miR-342-5p, miR-23b-3p, miR-338-3p, miR-3613-3p			
↑ miR-146a, miR-100, miR-505, miR-4467, miR-766, miR-3622b-3p, miR-296<br>↓ miR-449, miR-1274a, miR-4674, miR-335, miR-375, miR-708, miR-219, miR-103	CSF	AD patients	[249]
↑ miR-26b-3p, miR-28-3p, miR-30c-5p, miR-30d-5p, miR-148b-5p, miR-151a-3p, miR-186-5p, miR-425-5p, miR-550a-5p, miR-1468, miR-4781-3p, miR-5001-3p, miR-6513-3p<br>↓ let-7a-5p, let-7e-5p, let-7f-5p, let-7g-5p, miR-15a-5p, miR-17-3p, miR-29b-3p, miR-98-5p, miR-144-5p, miR-148a-3p, miR-502-3p, miR-660-5p, miR-1294, miR-3200-3p	Blood	AD patients	[187]
↓ miR-29c	Blood	AD patients	[160]
↑ miR-361-5p, miR-30e-5p, miR-93-5p, miR-15a-5p, miR-143-3p, miR-335-5p, miR-106b-5p, miR-101-3p, miR-424-5p, miR-106a-5p, miR-18b-5p, miR-3065- 5p, miR-20a-5p, miR-582-5p<br>↓ miR-1306-5p, miR-342-3p, miR-15b-3p	Serum	AD patients	[346]
↑ miR-125b, miR-222	CSF	AD patients	[353]
↑ miR-27b, miR-128, miR-155	PBMC	AD patients	[354]
↓ miR-9-5p, miR-106a-5p, miR-106b-5p, miR-107	Blood	AD patients	[355]
↑ miR-29a, miR-125b	CSF	AD patients	[356]

Supplementary Table S4. Brain miRNA regulation by physical exercise training.

	MicroRNA	Targets	Source	Type of Exercise	Reference
↑	miR-124	*BDNF, TRKB*	Brain	Treadmill running	[251]
↑	miR-504-5p<br>miR-103-3p<br>miR-669a-5p<br>miR-669p-5p<br>miR-449c-5p<br>miR-M1-6-3p<br>miR-21-3p<br>miR-667-5p<br>miR-208a-3p		Brain	Running wheel in traumatic brain injury	[357]
↓	miR-3089-3p<br>miR-34a<br>miR-23a-5p<br>miR-875-3p<br>miR-719<br>miR-m1-3-3p<br>miR-23-1-5p<br>miR-711<br>miR-M44-1<br>miR-295-5p				
↑	miR-28a-5p<br>miR-98a-5p<br>miR-148b-3p<br>miR-7a-5p<br>miR-15b-5p	Hdac5, Hdac6, SIRT-1	Brain	Exercise training in SAMP8 senescent mice<br>Running wheel	[246]
↓	miR-105<br>miR-133b-3p<br>miR-135a-5p<br>miR-203-3p<br>miR-190-5p				
↓	miR-21		Brain	Running wheel exercise in traumatic brain injury	[358]

↓ miR-124	*NR3C1* ↑	Brain	Rodent model of long-term voluntary exercise<br>Distance run	[238]
↑ miR-138<br>let-7c<br>miR-124<br><br>↓ miR-21<br>miR-92a<br>miR-874		Brain	Running wheel	[252]
↓ miR-34a	*DRP1* and *MFN2*	Brain	Swimming training in aging rats	[359]
↑ miR-132	BDNF and IGF-1 ↓	Brain	8 weeks os swimming training (rats) associated with genistein	[227]
↑ miR-129-1-3p<br>miR-144-5p<br>miR-10b-5p<br><br>↓ miR-708-5p	*IGFBP5, ITM2A* ↓<br><br><br><br>*CDKN1A, PER2, RTL-A2* ↑	Brain	4 weeks of treadmill exercise (rats)	[360]
↓ miR-132		Brain	SAMP8	[224]
↓ miR-1b	*BDNF*	Brain	Treadmill	[361]
↑ miR-96, miR-141, miR-182, miR-183, miR-200a, miR-200b, miR-200c, miR-263, miR-263a-5p, miR-429, miR-2881, miR-4510<br><br>↓ miR-7b, miR-34c, miR-84a, miR-122, miR-183, miR-448, miR-483, miR-1298, miR-1343, miR-2881, miR-3897-3p, miR-4154-3p, miR-4466, miR-4492, miR-4497, miR-4508, miR-4651, miR-5128		Brain	Swimming	[362]

⬇ miR-135a-5p, miR-190-5p, miR-203-3p	*IP3*	Brain	Running wheel	[363]
⬇ miR-137		Brain	Running wheel	[260]

Supplementary Table S5. Circulating miRNA regulation by physical exercise training.

MicroRNAs	Targets	Source	Models	Reference
⬆ miR-125a, miR-145, miR-181b, miR-193a, miR-197, miR-212, miR-223, miR-340, miR-365, miR-485, miR-505, miR-520d, miR-629, miR-638, miR-939, miR-940, miR-1225, miR-1238<br><br>⬇ miR-let-7i, miR-16, miR-17, miR-18a, miR-18b, miR-20a, miR-20b, miR-22, miR-93, miR-96, miR-106a, miR-107, miR-126, miR-130a, miR-130b, miR-151, miR-185, miR-194, miR-363, miR-660		Serum	Acute Response<br><br>Cycle ergometer exercise (10x2min bouts, 1min rest interval between each bout, 76% VO2peak	[364]
⬆ miR-21, miR-146a, miR-221, miR-222, miR-20a, miR-21, miR-146a, miR-221, miR-222	PTEN<br>PDCD4<br>p27/KIP1<br>p21/WAF1	Serum	Acute Response<br>Cardiopulmonary exercise test<br>Chronic Adaptation (90 days)<br>Rowing training, 5Km, 1-3 h per session, 20-24 strokes/min)	[264]
⬆ miR-7, miR-15a, miR-21, miR-26b, miR-132, miR-140, miR-181a, miR-181b, miR-181c, miR-338, miR-363, miR-939, miR-940, miR-1225<br><br>miR-let-7e, miR-23b, miR-31, miR-99a,		Serum	Acute Response<br><br>Cycle ergometer exercise (10x 2min bouts, 1min rest interval between each bout, 76% VO2peak)	[228]

↓	miR-125a, miR-125b, miR-126, miR-130a, miR-145, miR-151, miR-199a, miR-199b, miR-221, miR-320, miR-451, miR-486, miR-584, miR-652				
↑<br>↓	miR-149<br>miR-146a, miR-221		Serum	Acute Response<br>Resistance exercise (bench press and leg press)<br>3 days after exercise	[257]
↓	miR-486	PTEN	Serum	Acute Response<br>Cycle ergometry 60 min at 70% VO2max<br>Chronic Adaptation<br>(4 weeks total)<br>Systematic—cycling at 70% VO2max<br>(3 x30 min/ week)	[365]
↑<br>↓	miR-7, miR-29a, miR-29b, miR-29c, miR-30e, miR-142, miR-192, miR-338, miR-363, miR-590<br>miR-let-7e, miR-126, miR-130a, miR-151, miR-199a, miR-221, miR-223, miR-326, miR-328, miR-652		Serum	Acute Response<br>Cycle ergometer exercise (10x 2min bouts, 1min rest interval between each bout, 77% VO2peak)	[366]
↑	miR-181b, miR-214<br>miR-1, miR-133a, miR-133b, miR-208b		Plasma	Acute Response<br>Uphill treadmill test (concentric)<br>Immediately after<br>Downhill treadmill test	[367]

				(eccentric)<br>2-6 hs after exercise	
↑	miR-126, miR-133	CPK	Plasma	Acute Response<br>Single symptom-limited spiroergometry test<br>Cycling 4 h at 70% of anaerobic threshold<br>Marathon run<br>Eccentric resistance exercise	[368]
↑	miR-1, miR-126, miR-133a, miR-134, miR-146a, miR-208a, miR-499-5p	CPK<br>NT-proBNP<br>hsCRP	Plasma	Acute Response<br>Marathon run<br>Immediately after run<br>(decreased after 24 h)	[265]
↑	miR-1, miR-133a, miR-206, miR-208b, miR-499		Plasma	Acute Response<br>Marathon run<br>Immediately after run	[369]
↑	miR-1, -133a, -206		Plasma	Acute Response<br>Marathon run<br>Immediately after run	[370]
↑<br><br>↓	miR-15a, miR-29b, miR-29c, miR-30e, miR-140, miR-324, miR-338, miR-362, miR-532, miR-660<br><br>miR-23b, miR-130a, miR-151, miR-199a, miR-221		Serum	Acute Response<br><br>Cycle ergometer exercise (10x 2min bouts, 1min rest interval between each bout, 82% VO2max)	[371]
↑<br><br>↓	miR-1, miR-133a, miR-133b, miR-139-5p, miR-143, miR-145, miR-223, miR-330-3p, miR-338-3p, miR-424<br><br>miR-30b, miR-106a, miR-146, miR-151-3p, miR-151-5p, miR-221, miR-652, let-7i		Plasma	Acute Response<br>cycle ergometry test at 65% Pmax<br>1-3 hs after exercise<br><br>Immediately after exercise	[247]

↑ miR-103, miR-107,<br>↓ miR-21, miR-25, miR-29b, miR-92a, miR-133a, miR-148a, miR-148b, miR-185, miR-342-3p, miR-766, let-7d			Adaptation (12 weeks total) Systematic endurance cycle ergometry training, 3-5 days after training	
↑ miR-1, miR-133a, miR-133b, miR-206, miR-208b, miR-499		Plasma	Adaptation (5 months total) Systematic resistance training 36-72 hs after training	[372]
↑ let-7f, miR-21, miR-29c, miR-223<br>↓ miR-let-7f, miR-21, miR-29c, miR-223		Serum	Adaptation (18 weeks) Running exercise (3x/week, 60 min)	[373]
↑ miR-222	HIPK1	Plasma	Acute Response Heart failure patients Bicycle Ergometry Test	[374]
↑ let-7d-3p, let-7f-3p miR-29a-3p, miR-34a-5p, miR-125b-5p miR-132-3p, miR-143-3p, miR-148a-3p, miR-223-3p, miR-223-5p miR-424-3p, miR-424-5p		Serum	Acute Response Marathon run Immediately after run (decreased after 24 h)	[229]
↑ miR-1, miR-30a, miR-133a<br>↓ miR-26a, -29b		Plasma	Acute Response<br>Marathon run Immediately after run (decreased after 24 h)<br>Immediately after run	[375]

↑ miR-1, miR-133a, miR-206		Plasma	Acute Response<br>Marathon run<br>Immediately after run<br>(decreased after 24 h)	[376]
↑ miR-1, miR-133a,<br>miR-133b, miR-206<br>miR-485-5p, miR-509-5p,<br>miR-517a, miR-518f,<br>miR-520f, miR-522,<br>miR-553, miR-888	NF-κB	Plasma	Acute Response<br>High intensity interval<br>exercise<br>85-95% of HRmax<br>Immediately after<br>Vigorous intensity<br>continuous exercise<br>Immediately after	[377]
↑ miR-19a, miR-19b,<br>miR-20a, miR-26b,<br>miR-143, miR-195	p-AKT<br>p-S6K1	Serum	Acute Response<br>Resistance exercise<br>(3x bilateral knee extension<br>and leg press, 10 rep, 80%<br>of 1 RM)	[378]
↑ miR-1, miR-486, miR-494	HDAC4<br>PAX7<br>PTEN<br>FOXO1A	Serum	Acute Response<br>Aerobic exercise VO2max<br>test<br>(Endurance athletes,<br>runners, cyclists and<br>triathletes)	[379]
↑ miR-126	PI3KR2	Plasma	Adaptation<br>(10 weeks total)<br>Zucker rats<br>Swimming training<br>(60 min, 5d/ week)	[380]
↑ miR-376a<br>↓ miR-16, miR-27a, miR-28		Plasma	Adaptation<br>(5 months total)<br>Aerobic run exercise<br>training<br>30 min, 65-70% heart rate<br>res.<br>(4 days/week)	[381]

↑<br><br>↓	miR-21, miR-16, miR-93, miR-222<br><br>miR-222, miR-16		Plasma	Adaptation<br><br>8 weeks of: Explosive strength training, Hypertrophic strength training, High-intensity interval training	[382]
↑<br><br>↓	miR-221<br><br>miR-208b, miR-221, miR-21, miR-146a, miR-210		Serum	Acute Response and Adaptation<br><br>Basketball Exercise (3-months)	[258]
↑<br><br>↓	miR-21-5p, miR-27a-3p, miR-29a-3p, miR-30a-5p, miR-34a-5p, miR-126-3p, miR-132-3p, miR-142-5p, miR-143-3p, miR-150-5p, miR-195-5p, miR-199a-3p<br><br>miR-16-5p, miR-29b-3p, miR-30b-5p, miR-103a-3p, miR-106b-5p, miR-107, miR-139-3p miR-375, miR-497-5p, miR-590-5p		Serum	Acute Response<br><br>10 Km race, half-marathon, marathon	[230]
↑	miR-382	Resistin	Serum	Adaptation Swimming Training (12-weeks)	[253]
